# A cross sectional seroprevalence study of *Bartonella quintana* and *Bartonella henselae* among patients with human immunodeficiency virus (HIV) in Iran

**DOI:** 10.1016/j.nmni.2026.101841

**Published:** 2026-09-04

**Authors:** Mina Latifian, Malihe Hasannezhad, Ladan Abbasian, Zahra Tahmasebi Ashtiani, Mina Arabian, Fahimeh Bagheri Amiri, Saber Esmaeili

**Affiliations:** a Department of Epidemiology and Biostatistics, Research Centre for Emerging and Reemerging Infectious Diseases, Pasteur Institute of Iran, Tehran, Iran; b National Reference Laboratory of Plague, Tularemia and Q Fever, Research Centre for Emerging and Reemerging Infectious Diseases, Pasteur Institute of Iran, Akanlu, Kabudar Ahang, Hamadan, Iran; c Iranian Research Center for HIV/AIDS, Iranian Institute for Reduction of High Risk Behaviors, Department of Infectious Diseases, Imam Khomeini Hospital Complex, School of Medicine Tehran University of Medical Sciences, Tehran, Iran

**Keywords:** *Bartonella quintana*, *Bartonella henselae*, IFA, HIV, AIDS, Iran

## Abstract

**Background:**

*Bartonella henselae* and *Bartonella quintana* are among the most clinically relevant *Bartonella* species infecting humans. While *B. henselae* is a zoonotic pathogen maintained in animal reservoirs, *B. quintana* is considered a human-restricted bacterium transmitted via the human body louse. Both species can cause a wide range of manifestations, particularly in immunocompromised individuals such as those with HIV/AIDS. This study aimed to estimate the prevalence of IgG and IgM seropositivity to these two species and to identify determinants of seropositivity.

**Methods:**

This cross-sectional study included 200 participants, comprising 97 HIV-positive patients, 53 patients with AIDS, and 50 healthy individuals as the control group. IgG antibodies against *B. henselae* and *B. quintana* were measured in all participants using an indirect immunofluorescence assay (IFA). In addition, IgM antibodies were assessed in a randomly selected subset of 48 participants.

**Results:**

The overall seroprevalence of *B. henselae* and *B. quintana* IgG was 24.0% and 22.0%, respectively, while IgM seropositivity was 18.8% and 22.9%. No significant differences were observed in *B. quintana* IgG prevalence among control, HIV, and AIDS groups. Individuals with a history of drug abuse had a more than threefold increased likelihood of IgG positivity. For *B. henselae*, IgG prevalence was higher in the control and AIDS groups compared to HIV-positive individuals, though not statistically significant.

**Conclusion:**

The significant prevalence of IgG antibodies against both *Bartonella* species and the presence of IgM antibodies in the selected study group, especially in HIV patients, demonstrate the clinical importance of these pathogens in at-risk populations. These findings emphasize the need for clinical vigilance in symptomatic immunocompromised patients.

## 1 Introduction

*Bartonella* is a vector-borne, intracellular, Gram-negative bacterium that has been confirmed to cause infections in humans and animals. More than 45 species of this pathogen have been identified to date, with three species (*B. henselae*, *B. quintana*, and *B. bacilliformis*) implicated in human bartonellosis infections [1]. *Bartonella* species have been identified in a wide range of mammals and arthropod vectors [2]. The bacteria are transmitted by vectors such as lice, fleas, and ticks, as well as through scratches and bites from infected animals [3]. Fleas are considered the primary vectors for the maintenance and transmission of *Bartonella* species, feeding on mammalian hosts such as cats, dogs, rodents, insectivores, and rabbits [4].

Important diseases caused by species of this bacterium include cat scratch disease (caused by *B. henselae*), carrion disease (caused by *B. bacilliformis*), and trench fever (caused by *B. quintana*). Cat scratch disease, caused by *B. henselae*, is an infectious disease with symptoms ranging from mild to severe. Although in most patients the disease resolves spontaneously within 2 to 4 months without treatment, antibiotic therapy is recommended for patients with severe forms and/or those with suppressed immune systems, such as individuals with HIV/AIDS. Clinical manifestations may include maculopapular and papular skin lesions, lymphadenopathy, malaise, fatigue, headache, and occasionally fever. Rare or severe complications generally occur in people with immunodeficiency diseases [5].

Trench fever is caused by infection with *B. quintana*, which is primarily transmitted by the human body louse (*Pediculus* humanus). This disease most commonly affects homeless, alcoholic, and impoverished populations with inadequate sanitation and poor hygiene. *B. quintana* infection presents with a broad clinical spectrum, ranging from self-limited trench fever characterized by recurrent fever, headache, and severe leg pain to chronic bacteremia, culture-negative infective endocarditis, bacillary angiomatosis, bacillary peliosis, and persistent lymphadenopathy, particularly in immunocompromised individuals such as people living with HIV [6,7].

The host immune response plays a crucial role in preventing the development of systemic disease caused by *B. henselae* and *B. quintana*. Immunocompromised patients, including organ transplant recipients and HIV-infected individuals, are at higher risk of developing more severe disease [8]. The pathological response to Bartonella infection depends on the host's immune status: in immunocompetent individuals, it is typically granulomatous and suppurative, whereas in immunocompromised individuals, these bacteria can rapidly cause proliferative or vascular suppurative manifestations [9]. The cat flea, *Ctenocephalides felis*, serves as the primary vector for horizontal transmission of *B. henselae* among cats, and its bite can also transmit the bacteria to humans. Additionally, infection can occur through scratches, bites, or licking by an infected cat [10].

The first molecularly confirmed case of bacillary angiomatosis caused by *B. quintana* in Iran was reported in an HIV-infected patient in 2021 [11]. Additionally, outbreaks of *Bartonella* endocarditis, caused by *B. henselae* and *B. quintana*, have been documented in recent years [12]. More than eight different *Bartonella* species have also been identified in insects and livestock reservoirs in Iran, five of which are known to cause human disease. Therefore, *Bartonella* infections should be considered an important public health concern in the country, particularly among immunocompromised populations. In this context, the present study aimed to investigate the seroprevalence and risk factor of *Bartonella* species among patients with HIV/AIDS attending the Iranian AIDS Research Center.

## 2 Material and methods

**Ethics approval**: This study was conducted in accordance with the Declaration of Helsinki and received approval from the Human Ethics Committee of Tehran University of Medical Sciences (approval code: IR. TUMS.IKHC.REC.1402.377) for research involving human participants. All study procedures were additionally approved by the Committee of Imam Khomeini Hospital Complex, Tehran University of Medical Sciences, and were performed in accordance with the institution's relevant guidelines and regulations.

**Sampling:** In this study, to assess the serological prevalence of *B*. *henselae* and *B*. *quintana* infections, a total of 200 serum samples were collected between September 2023 and May 2024. The samples comprised 150 patients with HIV infection and 50 healthy individuals serving as controls. Patients were recruited from the Infectious Diseases Department and HIV clinic at Imam Khomeini Hospital, a referral university hospital in Tehran and samples were selected based on laboratory-confirmed CD4 T-cell count. Within the HIV-positive group, 39 individuals met the criteria for AIDS (CD4 T-cell counts below 200 cells/μL and/or clinical AIDS-defining conditions), whereas 111 HIV-infected patients with CD4 counts above 200 cells/μL were classified as non-AIDS HIV-positive. Control samples were randomly selected. Following informed consent, 5 mL of venous blood was collected from each participant into clot tubes for serological testing. A structured questionnaire was completed to obtain demographic data, medical history, history of animal contact and tick bites, social status, and history of blood product transfusions. All samples were transported to the Epidemiology and Biostatistics Department of the Pasteur Institute of Iran under cold chain conditions. Serum samples were subsequently separated and stored at −20 °C until serological analysis.

**Detection of specific IgG/IgM antibodies to *B. henselae* and *B. quintana*:** Due to safety requirements in working with HIV-positive samples, all sera were treated with Triton X-100 (1X) solution for 30 min at 37 °C before performing serological tests to reduce the risk of biocontamination [13]. Each serum sample was individually tested for *B. henselae* and *B. quintana*-specific IgG antibodies using a commercial indirect immunofluorescence assay (IFA) kit (Vircell, Granada, Spain). According to the manufacturer's instructions, each serum sample was serially diluted (1:64, 1:128, 1:256, up to 1:2048). A 5 μL aliquot of each dilution was applied to the designated wells of slides coated with fixed *B. henselae* and *B. quintana* antigens. Positive and negative controls provided in the kit were included in each run.

Following the initial incubation for 30 min at 37 °C, the slides were rinsed with phosphate-buffered saline (PBS) and immersed in PBS for 10 min. Slides were then briefly dipped in distilled water, air-dried at room temperature, and overlaid with 5 μL of fluorescein-labeled anti-human IgG conjugate. After a second incubation (30 min, 37 °C) and washing as described above, a mounting medium was applied, and coverslips were gently placed to seal the slides.

Fluorescence was examined using an indirect fluorescence microscope at 400× magnification. The highest serum dilution showing distinct apple-green fluorescence was recorded as the final antibody titer. Samples with titers above 1:2048 were further serially diluted and retested to determine the endpoint titer. A titer of ≥ 1:64 was considered positive for *Bartonella* IgG antibodies, as recommended by the manufacturer.

For the detection of *Bartonella*-specific IgM antibodies, the same sera (previously treated with Triton X-100) were tested individually using the Vircell IFA kit (Spain). Based on the manufacturer's protocol, each serum was first diluted 1:2 and mixed with IgG sorbent (10 μL serum + 50 μL IgG sorbent) to remove residual IgG antibodies. After centrifugation, the supernatant was used to prepare serial dilutions (1:12, 1:24, 1:48, and up to 1:1536). A 5 μL aliquot of each dilution was placed in the wells of antigen-coated slides containing fixed *B. henselae* and *B. quintana*. Positive and negative controls provided by the manufacturer were included in each assay.

Slides were incubated for 90 min at 37 °C, rinsed with PBS, immersed in PBS for 10 min, briefly dipped in distilled water, and air-dried. Then, 5 μL of fluorescein-labeled anti-human IgM conjugate was added to each well, followed by another incubation for 30 min at 37 °C. After washing, the slides were mounted and examined under an indirect fluorescence microscope (400× magnification).

The endpoint titer was defined as the highest dilution showing specific fluorescence. Samples with titers above 1:1536 were further diluted and retested to determine the final value. A titer of ≥ 1:24 was considered positive for *Bartonella* IgM antibodies, according to the manufacturer's recommendations.

**Statistical analysis**: Statistical analysis was performed using SPSS 26.0. Descriptive statistics were reported as mean ± SD and frequency (%). 95% CIs for seroprevalence were calculated using the Mid-P exact method. Normality was checked using the Kolmogorov–Smirnov test. Between-group comparisons were made using chi-square/Fisher's exact, *t*-test, or Mann–Whitney *U* test, as appropriate. Univariable and multivariable logistic regression (backward LR) were used to identify factors associated with seropositivity; variables with P < 0.2 entered the multivariable model. ORs with 95% CI were reported. All tests were two-tailed, with P < 0.05 considered significant.

## 3 Results

**Demographic, clinical, and laboratory data:** A total of 200 participants were enrolled in this study, including 150 individuals diagnosed with HIV/AIDS and 50 healthy controls (Table 1). The mean age was 44.80 years (SD: 11.43) in the AIDS group, 44.13 years (SD: 12.60) in the HIV-positive group, and 41.90 years (SD: 14.93) in the control group. Overall, the majority of participants were male (55.5%) and resided in urban areas (99.0%). All individuals in the AIDS and control groups were urban residents, while only one HIV-positive participant (1.0%) lived in a rural area.

**Table 1:** Demographic, clinical, and laboratory characteristics of the study participants.

		Control	AIDS	HIV+
**Age**	(Mean and SD)	41.9 (14.93)	44.8 (11.43)	44.13 (12.6)
**BMI**	(Mean and SD	25.9 (5.01)	22.7 (5.2)	26.1 (5.2)
**Gender**	Female	31 (62.0)	12 (26.1)	46 (44.2)
	Male	19 (38.0)	34 (73.9)	58 (55.8)
**Place**	Rural	0 (0.00)	0 (0.00)	1 (1.0)
	Urban	50 (100.0)	46 (100.0)	103 (99.0)
**Province**	Other	3 (6.0)	6 (13.04)	3 (2.88)
	Tehran/Alborz	47 (94.0)	40 (86.96)	101 (97.12)
**Education**	<12 years	5 (10.0)	28 (60.90)	45 (43.30)
	12-14 years	18 (36.0)	14 (30.40)	42 (40.40)
	14-16 years	14 (28.00)	3 (6.50)	13 (12.50)
	16-18 years	10 (20.00)	1 (2.20)	4 (3.80)
	>18 years	3 (6.00)	0 (0.00)	0 (0.00)
**Smoking**	Current smoker	4 (8.0)	22 (47.8)	31 (29.8)
	former smoker	1 (2.0)	7 (15.2)	8 (7.7)
	No	45 (90.0)	17 (37.0)	65 (62.5)
**Drug**	Yes	2 (4.00)	22 (48.90)	15 (14.40)
	No	48 (96.00)	23 (51.10)	89 (85.60)
**Alcohol**	Yes	5 (10.20)	11 (24.40)	12 (11.50)
	No	44 (89.80)	34 (75.60)	92 (88.50)
**Exposure to dog/cat**	Yes	5 (10.00)	21 (45.70)	27 (26.00)
	No	45 (90.00)	25 (54.30)	77 (74.00)
**Having pet**	Yes (Birds)	2 (4.0)	5 (10.9)	10 (9.6)
	Yes (Dog)	2 (4.0)	10 (21.7)	15 (14.4)
	Yes (Cat)	2 (4.0)	1 (2.2)	4 (3.8)
	No	44 (88.00)	16 (65.2)	29 (72.1)
**Exposure to farm animals**	Yes	1 (2.00)	4 (8.70)	2 (1.90)
	No	49 (98.00)	42 (91.30)	102 (98.10)
**Scratch with a cat**	Yes	4 (8.00)	6 (13.00)	9 (8.70)
	No	46 (92.00)	4 (87.00)	95 (91.30)
**Bite with ectoparasites**	Yes	0 (0.00)	1 (2.20)	1 (1.00)
	No	49 (100.00)	44 (97.80)	103 (99.00)
**Flea infestation in the last 5 years**	Yes	1 (2.00)	4 (8.90)	3 (2.90)
	No	49 (98.00)	41 (91.10)	101 (97.10)
**Exposure to Rat or Mice**	Yes	0 (0.00)	6 (13.30)	3 (2.90)
	No	49 (100.00)	39 (86.70)	100 (97.10)
**Income per month**	No income	9 (18.00)	14 (31.10)	39 (39.00)
	<71$	3 (6.00)	5 (11.10)	12 (12.00)
	71-143$	6 (12.00)	18 (40.00)	17 (17.00)
	>143$	32 (64.00)	8 (17.80)	32 (32.00)
**Homeless**	Yes	1 (2.00)	0 (0.00)	3 (2.900)
	No	49 (98.00)	45 (100.00)	100 (97.10)
**Underlying diseases**	Yes	14 (28.00)	14 (30.40)	20 (19.20)
	No	36 (72.00)	32 (69.60)	84 (80.80)
**Vaccine**	Yes	7 (14.30)	37 (82.20)	99 (95.20)
	No	42 (85.70)	8 (17.80)	5 (4.80)
**Received blood/plasma**	Yes	0 (0.0)	12 (26.7)	26 (25.0)
	No	49 (100.0)	33 (73.3)	78 (75.0)

In the AIDS group, females accounted for 26.1% and males for 73.9%, whereas in the HIV-positive group, 44.2% were female and 55.8% were male. Current smoking was most frequently reported among patients with AIDS (47.8%), followed by HIV-positive individuals (29.8%) and healthy controls (8.0%). Former smoking was reported in 15.2% of patients with AIDS, 7.7% of HIV-positive individuals, and 2.0% of controls. Alcohol consumption was more common in the AIDS group (24.4%) than in HIV-positive participants (11.5%) and controls (10.2%). A history of drug use was reported by 48.9% of AIDS patients, 14.4% of HIV-positive individuals, and 4.0% of controls.

A history of contact with dogs or cats was reported by 45.7% of patients with AIDS, compared with 26.0% of HIV-positive individuals and 10.0% of healthy controls. Similarly, cat scratches were reported by 13.0%, 8.7%, and 8.0% of participants in the AIDS, HIV-positive, and control groups, respectively. Homelessness was uncommon and was reported only among HIV-positive participants (2.9%), whereas no participants in the AIDS or control groups reported a history of homelessness (Table 1).

Regarding comorbidities, nearly one-quarter of HIV/AIDS patients (24.7%) had at least one underlying medical condition. Reported comorbidities included diabetes mellitus (4.0%), asthma (2.0%), respiratory disorders (0.7%), hepatitis (1.3%), cardiovascular disease (1.3%), hypertension (1.3%), hyperlipidemia (4.7%), thyroid disorders (2.7%), liver disease (0.7%), tuberculosis (1.3%), and a history of fungal infection (candidiasis) (0.7%). Furthermore, 72.2% of participants reported a history of vaccination, and 19.2% had previously received blood or blood products (Table 1).

**Seroprevalence of IgG and IgM antibodies against *Bartonella* species:** IgM testing was performed on a randomly selected subset of 48 participants (12 controls, 22 HIV-positive non-AIDS, and 14 AIDS patients), regardless of their IgG status. The overall seroprevalence of *B. henselae* and *B. quintana* IgG antibodies in the total study population (HIV/AIDS and controls combined) were 24.0% (95% CI: 18.5–30.3) and 22.0% (95% CI: 16.7–28.1), respectively. The overall seroprevalence of IgM antibodies were 18.8% (95% CI: 9.6–31.6) for *B. henselae* and 22.9% (95% CI: 12.7–36.3) for *B. quintana*.

**Seroprevalence of IgG and IgM antibodies against *B. quintana:*** The prevalence of *B. quintana* IgG was 26.0% in controls, 28.3% in AIDS patients, and 17.3% in HIV-positive non-AIDS individuals; these differences were not statistically significant. The prevalence of *B. quintana* IgM was 33.3% in controls, 28.6% in AIDS patients, and 13.6% in HIV-positive individuals; these differences were also not statistically significant.

In the univariable analysis for determinants of *B. quintana* IgG seropositivity, lower age, lower BMI, and drug abuse were identified as potential risk factors. or *B. quintana* seropositivity, the variables meeting this criterion and entered into the initial multivariable model were age, BMI category, history of drug use, and history of cat attack/scratch during the previous year. Backward likelihood-ratio (LR) selection was then applied. BMI category was removed during the backward selection procedure (P = 0.321), while age, history of drug use, and history of cat attack/scratch remained in the final model. In the multivariable analysis, higher age remained a protective factor (OR: 0.48, 95% CI: 0.24–0.97), and a history of drug use was significantly associated with increased odds of IgG seropositivity (OR: 3.28, 95% CI: 1.47–6.91). Participants reporting such exposure had higher odds of seropositivity than those without this history (aOR = 11.996, 95% CI: 1.406–102.321; P = 0.023), although the wide confidence interval indicates limited precision of the estimate. No other variables showed a statistically significant association with *B. quintana* IgG seropositivity (Table 2).

**Table 2:** Univariable analysis for assessing relationship between IgG and IgM positive results of *Bartonella quintana* and variables.

Variable		IgM Seropositive (n = 48) Positive, n (%)"	OR (95% CI)	P value	IgM Seropositive (n = 48) Positive, n (%)"	OR (95% CI)	P value
**Group**	Control	13 (26.0)	1	0.24	4 (33.3)	1	0.36
	AIDS	13 (28.3)	1.12 (0.46-2.76)		4 (28.6)	0.80 (0.15-4.25)	
	HIV+	18 (17.3)	0.60 (0.27-1.34)		3 (13.6)	0.32 (0.06-1.75)	
**Age**	<40	22 (30.1)	1	0.04	4 (19.0)	1	0.73
	≥40	22 (17.3)	0.49 (0.25-0.96)		7 (25.9)	1.45 (0.37-5.96)	
**BMI**	<18.5	8 (53.3)	1	0.02	1 (14.3)	1	0.88
	18.5-24.99	20 (23.0)	0.26 (0.08-0.81)		6 (27.3)	2.25 (0.22-22.80)	
	25-29.99	11 (16.7)	0.18 (0.05-0.58)		3 (23.1)	1.80 (0.15-21.48)	
	>30	5 (15.6)	0.16 (0.04-0.65)		1 (16.7)	1.20 (0.06-24.47)	
**Gender**	Female	1 (8.3)	1	0.58	0 (0.0)	ND	0.99
	Male	43 (22.9)	3.26 (0.41-25.97)		11 (23.9)	ND	
**Place**	Rural	0 (0.0)	ND		NA	ND	0.99
	Urban	44 (22.1)	ND		11 (22.9)	ND	
**Province**	Other	1 (8.3)	1	0.47	0 (0.0)	ND	
	Tehran/Alborz	43 (22.9)	3.26 (0.41-25.98)		11 (23.9)	ND	
**Academic Education**	No	18 (23.1)	1	0.77	7 (30.4)	1	0.24
	Yes	26 (21.3)	0.93 (0.46-1.89)		4 (16.0)	0.43 (0.11-1.75)	
**Smoking**	No	23 (18.3)	1	0.095	5 (19.2)	1	0.51
	Yes	21 (28.4)	1.77 (0.90-3.50)		6 (27.3)	1.58 (0.41-6.10)	
**Drug**	No	28 (17.5)	1	0.002	7 (21.9)	1	0.99
	Yes	16 (41.0)	3.28 (1.54-6.99)		4 (25)	1.19 (0.29-4.87)	
**Alcohol**	No	37 (21.8)	1	0.70	9 (20.9)	1	0.32
	Yes	7 (25)	1.20 (0.47-3.04)		2 (40.0)	2.52 (0.36-17.42)	
**Underlying diseases**	No	36 (23.7)	1	0.31	9 (22.0)	1	0.65
	Yes	8 (16.7)	0.64 (0.28-1.50)		2 (28.6)	1.42 (0.23-8.59)	
**Exposure to dog/cat**	No	33 (22.4)	1	0.80	9 (26.5)	1	0.36
	Yes	11 (20.8)	0.91 (0.42-1.95)		2 (14.3)	0.46 (0.09-2.48)	
**Having pet**	No	34 (22.8)	1	0.63	10 (27)	1	0.42
	Yes	10 (19.6)	0.83 (0.37-1.82)		1 (9.1)	0.27 (0.03-2.39)	
**Exposure to farm animal**	No	44 (22.8)	ND	0.35	11 (22.9)	1	0.99
	Yes	0 (0.0)	ND		0 (0.0)	ND	
**Scratch with cat**	No	43	1	0.08	11 (24.4)	1	
	Yes	1	ND		0 (0.0)	ND	
**Flea infestation in last 5 year**	No	43 (21.9)	1	0.40	10 (21.3)	ND	0.23
	Yes	1 (50.0)	3.56 (0.22-58.07)		1 (100.00)	ND	
**Flea bite in last years**	No	42 (22.0)	1	0.99	10 (21.7)	1	0.41
	Yes	2 (25.0)	ND		1 (50.0)	3.6 (0.21-62.8)	
**Exposure to Rat or Mice**	No	42 (22.3)	1	0.99	11 (23.4)	1	0.99
	Yes	2 (22.2)	0.99 (0.20-4.96)		0 (0.0)	ND	
**Homeless**	No	42 (21.6)	1	0.99	11 (23.9)	1	0.99
	Yes	1 (25.0)	1.21 (0.12-11.90)		0 (0.0)	ND	
**Vaccine**	No	13 (23.6)	1	0.69	3 (25.0)	1	0.70
	Yes	30 (21.0)	0.86 (0.41-1.80)		7 (20.0)	0.75 (0.16-3.52)	
**Received blood/plasma**	No	35 (21.9)	1	0.99	6 (15.8)	1	0.08
	Yes	8 (21.1)	0.95 (0.40-2.26)		4 (44.4)	4.27 (0.88-20.67)	

NA: No test was done for rural participants, ND: Not Define.

**NA:** No test was done for rural participants. **ND:** Not Defined.

For *B. quintana* IgM (tested in 48 randomly selected participants), the seroprevalence was 22.9% overall. Although the odds of IgM seropositivity were higher among individuals with a history of flea bite in the last year (OR: 3.6), the association was not statistically significant (95% CI: 0.21–62.8; P > 0.05). All other assessed variables, including age, BMI, smoking, drug use, alcohol consumption, animal contact, and homelessness, showed no statistically significant association with *B. quintana* IgM seropositivity (Table 2).

The distribution of *B. quintana* IgG titers among participants was as follows: a titer ≥1:64 was observed in 12.5% of the HIV/AIDS group and 12.0% of the control group; ≥1:128 in 6.6% of the HIV/AIDS group and 8.0% of the control group; ≥1:256 in 0.6% of the HIV/AIDS group and 4.0% of the control group; ≥1:512 in 0.0% of the HIV/AIDS group and 2.0% of the control group; and ≥1:1024 in 0.6% of the HIV/AIDS group and 0.0% of the control group.

For *B. quintana* IgM, among the 12 controls tested, 76.7% were negative and 23.3% were positive, with titers of 1:24 (7.2%), 1:192 (3.8%), and 1:384 (3.8%). Among the 36 HIV/AIDS patients tested, 60.8% were negative and 39.2% were positive for at least one *Bartonella* species. Among those positive for *B. quintana* IgM, titers of 1:24, 1:96, and 1:192 were each observed in 6.5% of cases, and a titer of 1:48 was observed in 2.8%.

**Seroprevalence of IgG and IgM antibodies against *B. henselae:*** The prevalence of *B. henselae* IgG was 30.0% in controls, 30.0% in AIDS patients, and 18.3% in HIV-positive non-AIDS individuals; these differences were not statistically significant. The prevalence of *B. henselae* IgM was 16.7% in controls, 28.6% in AIDS patients, and 13.6% in HIV-positive individuals; these differences were also not statistically significant.

For the analysis of *B. henselae* seropositivity (IgG), the candidate variables meeting this criterion were group, age, BMI category, underlying disease, smoking history, history of drug use, and occupational/living contact with farm animals. These variables were entered simultaneously into the multivariable model, followed by backward likelihood-ratio (LR) selection. In the multivariable analysis, higher BMI categories were significantly associated with lower odds of *B. henselae* IgG seropositivity compared to BMI <18.5: BMI 18.5–24.99 (OR: 0.25, 95% CI: 0.07–0.88), BMI 25–29.99 (OR: 0.16, 95% CI: 0.04–0.62), and BMI ≥30 (OR: 0.16, 95% CI: 0.04–0.62). A history of drug use was significantly associated with increased odds of *B. henselae* IgG seropositivity (OR: 2.46, 95% CI: 1.09–5.59). Additionally, the presence of underlying diseases was significantly associated with lower odds of IgG seropositivity (OR: 0.31, 95% CI: 0.11–0.86).

For *B. henselae* IgM, no statistically significant associations were found with any of the assessed variables, including age, BMI, gender, smoking, drug use, animal contact, or flea bite (all P > 0.05; Table 3).

**Table 3:** Univariable analysis for assessing relationship between IgG and IgM positive results of *Bartonella*henselae and variables.

Variable		IgG Seropositive (n = 200) Positive, n (%)	OR (95% CI)		IgM Seropositive (n = 48) Positive, n (%)	OR (95% CI)	
Group	Control	15 (30.0)	1	0.14	2 (16.7)	1	0.52
	AIDS	14 (30.4)	1.02 (0.43-2.44)		4 (28.6)	2.00 (0.30-13.51)	
	HIV+	19 (18.3)	0.52 (0.24-1.14) NS		3 (13.6)	0.79 (0.12-5.53)	
**Age**	<40	22 (30.1)	1	0.12	3 (14.3)	1	0.71
	≥40	26 (20.5)	0.60 (0.31-1.16)		6 (22.2)	1.7 (0.37-7.9)	
**BMI**	<18.5	8 (53.3)	1	0.01	2 (28.6)	1	0.63
	18.5-24.99	24 (27.6)	0.33 (0.11-1.02)		3 (13.6)	0.40 (0.05-3.04)	
	25-29.99	11 (16.7)	0.18 (0.05-0.58) *		2 (15.4)	0.46 (0.05-4.22)	
	>30	5 (15.6)	0.16 (0.04-0.65) *		2 (33.3)	1.25 (0.12-13.24)	
**Gender**	Female	19 (21.3)	1	0.43	4 (23.5)	1	0.70
	Male	29 (26.1)	0.77 (0.40-1.49)		5 (16.1)	1.60 (0.37-5.99)	
**Place**	Rural	0 (0.0)	ND	0.99	NA	ND	
	Urban	48 (24.1)	ND		9 (18.8)	ND	
**Province**	Other	2 (16.7)	1	0.73	0 (0.0)	ND	0.99
	Tehran/Alborz	46 (24.5)	1.62 (0.34-7.66) NS		9 (19.6)	ND	
**Academic Education**	No	20 (25.6)	1	0.66	5 (21.7)	1	0.72
	Yes	28 (23.0)	0.87 (0.45-1.67)		4 (16.0)	0.69 (0.16-2.95)	
**Smoking**	No	26 (20.6)	1	0.15	3 (11.5)	1	0.27
	Yes	22 (29.7)	1.63 (0.84-3.15)		6 (27.3)	2.88 (0.63-13.22)	
**Drug**	No	31 (19.4)	1	0.002	5 (15.6)	1	0.46
	Yes	17 (43.6)	3.22 (1.53-6.77)		4 (25.0)	1.8 (0.41-7.91)	
**Alcohol**	No	41 (24.1)	1	0.76	8 (18.6)	1	0.99
	Yes	6 (21.4)	0.86 (0.33-2.26)		1 (20.0)	1.09 (0.11-11.15)	
**Underlying diseases**	No	42 (27.6)	1	0.03	7 (17.1)	1	0.60
	Yes	6 (12.5)	0.38 (0.15-0.95)		2 (28.6)	1.94 (0.31-12.12)	
**Exposure to dog/cat**	No	36 (24.5)	1	0.79	7 (20.6)	1	0.99
	Yes	12 (22.6)	0.91 (0.43-1.91) NS		2 (14.3)	0.64 (0.12-3.56)	
**Having pet**	No	39 (26.2)	1	0.22	9 (24.3)	ND	0.10
	Yes	9 (17.6)	0.61 (0.27-1.36) NS		0 (0.0)	ND	
**Exposure with farm animal**	No	48 (24.9)	ND	0.13	9 (18.8)	ND	ND
	Yes	0 (0.0)	ND		0 (0.0)	ND	
**Scratch with cat**	No	45 (24.9)	1	0.38	9 (20.0)	ND	0.99
	Yes	3 (15.8)	0.57 (0.16-2.04)		0 (0.0)	ND	
**Flea infestation in last 5 year**	No	47 (24.0)	1	0.43	8 (17.0)	ND	0.19
	Yes	1 (50.0)	3.17 (0.19-51.67)		1 (100.0)	ND	
**Flea bite in last years**	No	46 (24.1)	1	0.99	8 (17.4)	1	0.34
	Yes	2 (25.0)	1.05 (0.21-5.39)		1 (50.0)	4.75 (0.27-84.18)	
**Exposure to Rat or Mice**	No	47 (25.0)	1	0.69	9 (19.1)	ND	0.99
	Yes	1 (11.1)	0.38 (0.05-3.08)		0 (0.0)	ND	
**Homeless**	No	46 (23.7)	1	0.99	8 (17.4)	ND	0.99
	Yes	1 (25.0)	1.07 (0.11-10.57)		0 (0.0)	ND	
**Vaccine**	No	15 (27.3)	1	0.46	2 (16.7)	1	0.99
	Yes	32 (22.4)	0.77 (0.38-1.57)		7 (20.0)	1.25 (0.22-7.05)	
**Received blood/plasma**	No	39 (24.4)	1	0.83	6 (15.8)	1	0.34
	Yes	8 (21.1)	0.8 (0.35-1.96) NS		3 (33.3)	2.67 (0.52-13.71)	

**NA:** No test was done for rural participants. **ND:** Not Defined.

Data are presented as n (%). OR, odds ratio; CI, confidence interval.

The distribution of *B. henselae* IgG titers among participants was as follows: a titer ≥1:64 was observed in 14.0% of the HIV/AIDS group and 10.0% of the control group; ≥1:128 in 15.0% of the HIV/AIDS group and 8.0% of the control group; ≥1:512 in 0.6% of the HIV/AIDS group and 0.0% of the control group; and ≥1:1024 in 2.0% of the HIV/AIDS group and 0.5% of the control group.

For *B. henselae* IgM, among the 12 controls tested, 83.3% were negative and 16.7% were positive, with titers of 1:192 and 1:768 each observed in one participant (8.3%). Among the 36 HIV/AIDS patients tested, 19.4% had positive IgM titers, including 13.9% with a titer of 1:192, 2.8% with 1:96, and 2.8% with 1:384. No significant association was observed between louse bites and seropositivity for either species.

**Seropositivity in patients with low CD4 counts:** Among the 34 patients with low CD4 counts (<200 cells/μL), 28 (82%) were seropositive for at least one *Bartonella* antibody class (IgG and/or IgM). Although this finding indicates a high rate of previous or recent exposure to *Bartonella* spp., serological data alone cannot establish a clinically relevant infection or infer a causal association with immunodeficiency.

**Demographic, clinical, and laboratory data**: A total of 200 participants were enrolled in this study, including 150 individuals diagnosed with HIV/AIDS and 50 subjects in the control group (Table 1). The mean age was 44.80 years in the AIDS group and 44.13 years in the HIV-positive group. Overall, the majority of participants were male (55.5%) and resided in urban areas. All individuals in the AIDS group were urban residents, while only one participant (1.0%) in the HIV-positive group lived in a rural area.

In the AIDS group, females accounted for 26.1% of participants and males for 73.9%, whereas in the HIV-positive group, 44.6% were female and 55.8% were male. Current smoking was reported by 47.8% of individuals with AIDS, and 15.2% were former smokers.

Current smoking was most frequently reported among patients with AIDS (47.8%), followed by HIV-positive individuals (29.8%) and healthy controls (8.0%). Former smoking was reported in 15.2% of patients with AIDS, 7.7% of HIV-positive individuals, and 2.0% of controls. Alcohol consumption was also more common in the AIDS group (24.4%) than in HIV-positive participants (11.5%) and controls (10.2%).

A history of contact with dogs or cats was reported by 45.7% of patients with AIDS, compared with 26.0% of HIV-positive individuals and 10.0% of healthy controls. Similarly, cat scratches were reported by 13.0%, 8.7%, and 8.0% of participants in the AIDS, HIV-positive, and control groups, respectively. Homelessness was uncommon and was reported only among HIV-positive participants (2.9%), whereas no participants in the AIDS or control groups reported a history of homelessness (Table 1). Regarding comorbidities, nearly one-quarter of HIV/AIDS patients (24.7%) had at least one underlying medical condition. Reported comorbidities included diabetes mellitus (4.0%), asthma (2.0%), respiratory disorders (0.7%), hepatitis (1.3%), cardiovascular disease (1.3%), hypertension (1.3%), hyperlipidemia (4.7%), thyroid disorders (2.7%), liver disease (0.7%), tuberculosis (1.3%), and a history of fungal infection (candidiasis) (0.7%). Furthermore, 72.2% of participants reported a history of vaccination, and 19.2% had previously received blood or blood products (Table 1).

IgM testing was performed on a randomly selected subset of 48 participants who were testing for *B. henselae and B. quintana* IgG, including 12 controls, 19 HIV-positive individuals, and 17 AIDS patients. The prevalence of *B. henselae* and *B. quintana* for IgG were 24.0% (95% CI: 18.5-30.28) and 22.0% (95% CI: 16.7-28.1) in HIV/AIDS and control group, and the prevalence of IgM were 18.8% (95% CI: 9.6-31.6) and 22.9% (95% CI: 12.7-36.3) respectively.

**Seroprevalence of IgG and IgM antibodies against *B. quintana***: The prevalence of *B. quintana* IgG in control (26.0%), AIDS (28.3%) and HIV positive (17.3%) were not significantly different. The prevalence of *B. quintana* IgM in control (33.3%), AIDS (28.6%) and HIV positive (13.6%) were not significantly different. In the assessment of determinants of IgG positive result of *B. quintana*, lower age, lower BMI, and drug abuse were found as risk factors. In multivariable analysis, and after adjusting higher age were considered as a protective factor (OR: 0.48, 95% CI: 0.24-0.97). Also, the chance of IgG positive result in people with a history of drug abuse was 3.28 times (95% CI: 1.47-6.91) than people without history. IgM was tested in 48 people randomly including 12 controls, 14 AIDS patients, and 22 HIV positive individuals, and 22.9% of them were positive for *B. quintana* IgM. There was a higher odd of IgM seropositivity in people with a history of flea bite in the last year (OR: 3.6, 95% CI: 0.21-62.8) (Table 2). Although the odds of *B. quintana* IgM seropositivity was higher among individuals with a history of flea bite in the last year, the association was not statistically significant (OR: 3.6, 95% CI: 0.21-62.8; P > 0.05).

*B. quintana* IgG titers among participants were distributed as follows: a titer ≥1:64 was observed in 12.5% of the HIV/AIDS group and 12% of the control group; ≥1:128 in 6.6% of the HIV/AIDS group and 8% of the control group; ≥1:256 in 0.6% of the HIV/AIDS group and 4% of the control group; ≥1:512 in 0% of the HIV/AIDS group and 2% of the control group; and ≥1:1024 in 0.6% of the HIV/AIDS group and 0% of the control group. The IFA IgM test for *B. quintana* in the control group (n:12), showed that 76.6% were negative and 23.3% were positive. Among the positive cases, 7.16% had a titer of 1:24, 3.8% had a titer of 1:192, and 3.8% had a titer of 1:384. In the HIV/AIDS patient group, of the 36 patients tested, 60.8% were negative for both *Bartonella* species. Among the *B. quintana*-positive cases, 6.5% had a titer of 1:24, 6.5% a titer of 1:96, 6.5% a titer of 1:192, and 2.8% a titer of 1:48.

**Seroprevalence of IgG and IgM antibodies against *B. henselae:*** *B. henselae* IgG in control (30.0%) and AIDS (30.0%) were higher than HIV positive (18.3%) but it wasn't statistically significant. *B. henselae* IgM in control (16.7%) and AIDS (28.6%) were higher than HIV positive (13.6%) but it wasn't statistically significant. People with higher BMI had lower chance for IgM positive result for *B. henselae*. People with history of drug using were significantly positive for IgG of *B. henselae*. People with underlying disease had less chance for being IgG positive. After adjusting, BMI and drug using still remained as determinant factors. As all BMI groups had lower chance for being IgG positive rather than BMI lower than 18.5.18.5-24.99 (OR: 0.25, 95% CI: 0.70-0.88), BMI25-29.99 (OR: 0.16, 95% CI: 0.04-0.62), BMI>30 (OR: 0.16, 95% CI: 0.04-0.62). Also, people with underlying disease had lower chance of a positive result (0.31, 95% CI: 0.11-0.86). Individuals with a history of drug use had significantly higher odds of *B. henselae* IgG seropositivity than those without a history of drug use (OR: 2.46, 95% CI: 1.09–5.59) (Table 3)The distribution of *B. henselae* IgG titers among participants was as follows: a titer ≥1:64 was observed in 14% of the HIV/AIDS group and 10% of the control group; ≥1:128 in 15% of the HIV/AIDS group and 8% of the control group; ≥1:512 in 0.6% of the HIV/AIDS group and 0% of the control group; and ≥1:1024 in 2% of the HIV/AIDS group and 0.5% of the control group. Among the 12 controls tested, 10 (83.3%) were IgM-negative and 2 (16.7%) were IgM-positive, with titers of 1:192 and 1:768 observed in one participant each (8.3%). In the HIV/AIDS patient group (n:36), 19.4% had positive IgM titers, including 13.9% with a titer of 1:192, 2.8% with 1:96, and 2.8% with 1:384. No significant association was observed between louse bites and seropositivity.

Among the 34 patients with low CD4 counts (<200), 28 (82%) were seropositive for at least one *Bartonella* antibody class (IgG and/or IgM). Although this finding indicates previous or recent exposure to *Bartonella* spp., serological data alone cannot establish a clinically relevant infection or infer a causal association with immunodeficiency.

## 4 Discussion

In this study, including HIV-infected patients and healthy controls, the overall seroprevalence of IgG antibodies against *B. henselae* and *B. quintana* among all 200 participants was 24.0% and 22.0%, respectively, while the corresponding IgM seroprevalence was 18.8% and 22.9%. These findings indicate substantial exposure to *Bartonella* spp. in the study. population. The first HIV-infected patient with bartonellosis was identified in 1983 [14]. Subsequent studies have reported a seroprevalence of antibodies against *Bartonella* spp. among HIV-infected individuals ranging from 17.3% to 40% [15]. In a seroprevalence study conducted in Spain using IFA, 22.3% of HIV-infected patients tested positive for at least one *Bartonella* antibody [16], which aligns with the findings of our study. We also assessed *Bartonella* seroprevalence in a control group, with average IgG seroprevalence of 30% for *B. henselae* and 26% for *B. quintana*. In contrast, other studies have reported seroprevalence rates of 8.6% in healthy populations, which differs from our results [17]. Overall, bartonellosis appears to be relatively common in the general population, with *Bartonella* seroreactivity ranging from 2% to 30% in European and American cohorts [18]. Despite the recognized risk of bartonellosis among HIV-positive individuals, data on seroprevalence in this high-risk group remain limited. For instance, in South Africa, molecular testing revealed bartonellosis in 10% of HIV-positive outpatients, underscoring the need for ongoing surveillance in this vulnerable population [19].

In our study, a higher proportion of positive cases was observed among men (24.5%) compared to women (16.7%), whereas a study conducted in Spain reported the opposite, with most positive cases among women (24.4%). Additionally, we found a significant association between younger age and increased seropositivity, while in the Spanish study, the probability of seropositivity increased with age. Drug use was also directly associated with higher seropositivity in our study, consistent with previous finding [16]. While some studies have reported significant associations between *Bartonella* seropositivity in HIV-infected individuals and a history of animal contact or body lice exposure, particularly among homeless populations [20,21], we did not observe a significant relationship between seropositivity and either body lice or animal contact, including cats.

In a study conducted in Brazil, the seroprevalence of IgG antibodies to *Bartonella* among HIV-infected individuals was 40.8% [22]. In a study in the United States, 17% of 382 HIV-positive patients tested positive for *Bartonella* antibodies, with *B. quintana* showing a higher rate of seropositivity compared to *B. henselae*, which aligns partially with our findings [21]. In South Africa, the prevalence of IgG and IgM antibodies to *Bartonella* species among HIV-infected patients was reported as 32% and 14%, respectively, while in the control group it was 19% and 17%, respectively. Furthermore, in this study, *B. henselae* antibodies were more prevalent than those against *B. quintana*, which is consistent with the observations of our study [23].

The relatively higher seroprevalence observed among healthy controls in our study compared with some previous reports may reflect differences in environmental and epidemiological conditions rather than true inconsistencies in infection risk. Iran has a large rural population with frequent exposure to domestic animals, cats, dogs, and their ectoparasites, all of which may increase the likelihood of asymptomatic *Bartonella* exposure. In addition, variations in study populations, occupational characteristics, geographical settings, and serological assays (including antigen preparation and antibody titer cut-offs) may substantially influence reported seroprevalence estimates. Therefore, direct comparisons between studies should be interpreted with caution [1,7,24].

Our findings are consistent with a recent molecular study from Iran, which reported the first PCR-confirmed detection of *B. quintana* exclusively among HIV-positive and AIDS patients. Together, these molecular and serological findings support the circulation of *B. quintana* in immunocompromised individuals in Iran and highlight the complementary value of combining PCR with serological testing [25].

The diagnosis of *Bartonella* infection is generally challenging, particularly in asymptomatic individuals. Immunocompromised populations are at a heightened risk of exposure, and among HIV patients, those with CD4 counts below 50 cells/μL are particularly susceptible to severe disease and chronic opportunistic infections [26]. In our study, 34 patients exhibited low CD4 counts (<200), of whom 28 (82%) were seropositive for at least one antibody class (IgG or IgM). However, some studies have not observed a direct inverse relationship between CD4 count and seropositivity. For instance, a study in Spain reported that only 9.2% of patients with CD4 counts <200 (n = 47) tested positive for *Bartonella* antigens, with the majority of positive cases occurring in individuals with CD4 counts >200 [16].

The observed association between lower BMI and higher seropositivity for *Bartonella* spp. may reflect underlying immunological and socioeconomic factors. Underweight status has been previously associated with impaired cellular and humoral immune responses, particularly in populations affected by chronic infections, substance abuse, or malnutrition. Such immune dysfunction may facilitate persistent or repeated exposure to *Bartonella* species and enhance antibody detectability. In addition, low BMI has been reported as a marker of vulnerability in marginalized populations, including individuals with substance use disorders and unstable living conditions, which are known risk factors for exposure to ectoparasites such as lice and fleas the primary vectors of *B. quintana*. Similar associations between undernutrition and increased susceptibility to vector-borne or opportunistic infections have been described in previous studies among HIV-infected and homeless populations. Furthermore, chronic inflammation and immune activation in underweight individuals may contribute to prolonged antibody persistence, potentially explaining the higher IgG seroprevalence observed in this group. Our findings are consistent with previous reports indicating that nutritional status plays an important role in host susceptibility to *Bartonella* infections and serological outcomes [[27], [28], [29], [30]].

Microbiological detection of *Bartonella* infection is often limited by low sensitivity, and the intracellular nature of the bacteria can delay detection. Therefore, serological detection of antibodies is a valuable diagnostic tool. The most widely used method is the immunofluorescence assay (IFA) for antibodies against *B. henselae* and *B. quintana*, as recommended by the US Centers for Disease Control and Prevention. An anti-*B. henselae* IgG titer of ≥1:64 is considered indicative of previous exposure or supportive evidence when interpreted together with clinical findings. However, positive serology alone only suggests exposure rather than clinically relevant infection [31]. Cross-reactivity between *Bartonella*, *Coxiella*, and *Chlamydia* antigens may reduce the specificity of serological tests, which have reported sensitivities ranging from 50% to 95% [8]. PCR offers a rapid and specific approach for direct detection from clinical specimens and demonstrates higher sensitivity compared to culture methods [32]. Regarding treatment, beta-lactams were historically used, but current evidence supports the use of macrolides (erythromycin, azithromycin, clarithromycin), tetracyclines (particularly doxycycline), and rifampin as having the most reliable minimum inhibitory concentration (MIC) data and clinical efficacy [31].

One limitation of this study is that *Bartonella* seroprevalence was assessed solely using serological assays (IgG and IgM), without molecular confirmation by PCR. While this approach is appropriate for seroepidemiological studies in asymptomatic populations, it limits the ability to precisely differentiate between past and recent exposure. IgG seropositivity likely reflects previous exposure, whereas IgM positivity may suggest more recent exposure; however, this distinction cannot be definitively confirmed in the absence of molecular data. Because IgM antibodies may appear before detectable IgG seroconversion during early primary infection, this strategy may have failed to identify a small number of recent *Bartonella* infections. Consequently, the prevalence of recent infections reported in the present study may have been slightly underestimated. In addition, serological results may be influenced by potential cross-reactivity with other bacteria, including *Mycobacterium* species, which are highly prevalent among HIV-infected individuals.

An additional limitation of serological testing should be considered in HIV-infected individuals, particularly those with advanced immunosuppression. Impaired humoral immune responses in patients with low CD4^+^ T-cell counts may reduce antibody production, leading to false-negative or lower antibody titers despite active or recent *Bartonella* infection. Consequently, serological findings should be interpreted cautiously in this population and, whenever feasible, complemented by molecular methods such as PCR to improve diagnostic accuracy. Therefore, negative serological results do not completely exclude *Bartonella* infection in severely immunocompromised patients [33,34]. Additionally, some epidemiological exposure data, such as cat contact, lice or mite exposure, and rodent presence in residential areas, were missing for a subset of participants. The extent of missing data varied across variables and study groups, limiting the assessment of potential infection sources and their association with serological outcomes. Given the challenges of diagnosing *Bartonella* infection, particularly in immunocompromised individuals such as those with HIV, where bacteremia may be intermittent, and the use of standardized diagnostic approaches combining serological and molecular methods is critically important. Considering the limited seroepidemiological data on *Bartonella* infection among HIV-positive individuals in Iran, increased attention from the healthcare community is warranted to improve surveillance and management of this vulnerable population.

## 5 Conclusion

These findings suggest that exposure to *Bartonella* spp. occurs in both HIV-infected and non-infected populations; however, further studies are required to clarify the epidemiological and clinical significance of these findings. Our results highlight the need for increased clinical awareness of *Bartonella* exposure, particularly in immunocompromised individuals.

## CRediT authorship contribution statement

**Mina Latifian:** Writing – original draft, Methodology, Investigation, Formal analysis. **Malihe Hasannezhad:** Writing – review & editing, Project administration, Funding acquisition, Conceptualization. **Ladan Abbasian:** Writing – review & editing, Methodology, Data curation. **Zahra Tahmasebi Ashtiani:** Writing – original draft, Methodology, Investigation. **Mina Arabian:** Formal analysis, Software, Writing – review & editing. **Fahimeh Bagheri Amiri:** Writing – review & editing, Formal analysis, Data curation. **Saber Esmaeili:** Visualization, Validation, Resources, Project administration, Conceptualization.

## Funding

This study was supported by financial grants from Iranian Research Center for HIV/AIDS, 10.13039/501100004484Tehran University of Medical Sciences (Grant No. 6880-119-3-1402), and the 10.13039/501100010679Pasteur Institute of Iran (Grant No. 2362).

## Declaration of competing interest

The authors declare that they have no known competing financial interests or personal relationships that could have appeared to influence the work reported in this paper.
